# Direct and possible indirect effects of vaccination on rotavirus hospitalisations among children in Malawi four years after programmatic introduction

**DOI:** 10.1016/j.vaccine.2018.04.030

**Published:** 2018-11-12

**Authors:** A. Bennett, L. Pollock, K.C. Jere, V.E. Pitzer, U. Parashar, J.E. Tate, R.S. Heyderman, C. Mwansambo, N. French, O. Nakagomi, M. Iturriza-Gomara, D. Everett, N.A. Cunliffe, N. Bar-Zeev, for the VacSurv Consortium

**Affiliations:** a Malawi-Liverpool-Wellcome Trust Clinical Research Programme, College of Medicine, University of Malawi, Blantyre, Malawi; b Centre for Global Vaccine Research, Institute of Infection & Global Health, University of Liverpool, Liverpool, UK; c Department of Epidemiology of Microbial Diseases, Yale School of Public Health, Yale University, New Haven, CT, USA; d Centers for Disease Control and Prevention, Atlanta, USA; e Division of Infection and Immunity, University College London, UK; f Ministry of Health, Lilongwe, Malawi; g Department of Molecular Epidemiology, Nagasaki University, Nagasaki, Japan; h NIHR Health Protection Research Unit in Gastrointestinal Infections, University of Liverpool, Liverpool, UK

**Keywords:** Rotavirus, Indirect effects, Vaccines

## Abstract

**Introduction:**

Despite increased use of vaccine in routine immunisation, rotavirus remains a major cause of acute gastroenteritis (AGE) in low-income countries. We describe rotavirus prevalence and hospitalisation in Malawi pre and four years post vaccine introduction; provide updated vaccine effectiveness (VE) estimates; and assess rotavirus vaccine indirect effects.

**Methods:**

Children under five years of age presenting to a referral hospital in Blantyre with AGE were recruited. Stool samples were tested for rotavirus using Enzyme Immunoassay. The change in rotavirus prevalence was evaluated using Poisson regression. Time series analysis was used to further investigate trends in prevalence over time. VE against rotavirus diarrhoea of any severity was estimated using logistic regression. Indirect effects were estimated by evaluating rotavirus prevalence in unvaccinated children over time, and by comparing observed reductions in incidence of rotavirus hospitalisation to those expected based on vaccine coverage and trial efficacy estimates.

**Results:**

2320 children were included. Prevalence of rotavirus in hospitalised infants (<12 months) with AGE decreased from 69/139(49.64%) prior to vaccine introduction to 197/607(32.45%) post-vaccine introduction (adjusted RR 0.67[95% CI 0.55, 0.82]). Prevalence in children aged 12–23 months demonstrated a less substantial decline: 15/37(40.54%) pre- and 122/352(34.66%) post-vaccine introduction (adjusted RR 0.85, 95% CI 0.57, 1.28). Adjusted VE was 61.89%(95% CI 28.04–79.82), but lower in children aged 12–23 months (31.69% [95% CI −139.03 to 80.48]). In hospitalised infants with rotavirus disease, the observed overall effect of the vaccine was 9% greater than expected according to vaccine coverage and efficacy estimates. Rotavirus prevalence among unvaccinated infants declined post-vaccine introduction (RR 0.70[95% CI 0.55–0.80]).

**Conclusions:**

Following rotavirus vaccine introduction in Malawi, prevalence of rotavirus in hospitalised children with AGE has declined significantly, with some evidence of an indirect effect in infants. Despite this, rotavirus remains an important cause of severe diarrhoea in Malawian children, particularly in the second year of life.

## 1 Introduction

Prior to widespread routine vaccination, rotavirus was the commonest cause of childhood diarrhoea worldwide and in 2008 was estimated to be responsible for over 450,000 annual deaths [1]. Following World Health Organization (WHO) recommendation in 2009 [2], rotavirus vaccine has been introduced in 81 countries, including 37 low- and middle-income countries (LMICs) eligible for support from Gavi: the Vaccine Alliance [3]. In pre-licensure trials, substantially lower vaccine efficacy was observed in LMICs (39–72%) compared with high-income countries (98–100%) [4], [5], [6], [7], [8], [9]; however, post-introduction vaccine effectiveness estimates from LMICs have been encouraging (60–70%) [10], [11], [12], [13]. In the context of continued global roll-out of rotavirus vaccine, it is important to describe persistence of population impact and to quantify the residual burden of rotavirus disease, particularly amongst young children hospitalised with gastroenteritis.

Despite high levels of vaccine uptake (90–98%), in several LMICs over 20% of hospitalised gastroenteritis remains attributable to rotavirus [11], [13], [10]. In high-income settings, vaccine effectiveness (VE) appears to be sustained in the second year of life [14], but reports of persistence of protection in LMICs are mixed [15]. Some LMICs, including Malawi, have reported lower effectiveness estimates in the second year of life [11], [16], while others have observed sustained protection [15], [17]. In addition to the direct effects of vaccination, the indirect effects of rotavirus vaccine may contribute substantially to the overall population impact, particularly in countries where VE is suboptimal. Evidence of indirect effects from well-resourced settings exists [18], but data describing indirect effects from LMICs are lacking [13]. Because of differences in factors such as population density and patterns of contact that could affect the force of infection, the indirect effects of vaccination in LMICs could differ from well-resourced settings [19].

Malawi introduced monovalent rotavirus vaccine (RV1) on 29 October 2012, with doses at 6 and 10 weeks of life. We have previously published VE estimates and population impact 3 years after vaccine introduction [11], [12]. In this prospective observational study, we aimed to describe the prevalence of rotavirus hospitalisation in urban Blantyre, Malawi, 4 years after vaccine introduction, update published vaccine effectiveness estimates, and investigate for evidence of rotavirus vaccine indirect effects.

## 2 Methods

This study was conducted at Queen Elizabeth Central Hospital (QECH), Blantyre, Malawi. QECH is the only government hospital providing free inpatient care to the estimated 151,328 under-5 population of Blantyre City [11]. Active surveillance for acute gastroenteritis has been conducted at QECH since 1997; this paper describes the period of enhanced surveillance commencing 1 January 2012 to 30 June 2016.

Our surveillance platform has been described previously in detail [11], [12]. Briefly, children presenting to QECH with acute gastroenteritis (AGE) during routine clinical hours are enrolled following informed consent. Detailed demographic and clinical data are recorded, anthropometric assessment undertaken and bulk stool sample collected. HIV testing is conducted according to national guidance [20]. Vaccine status is obtained from government-issued family-held records. Disease severity is defined using Vesikari score [21]; a score of ≥11/20 indicates severe disease. HIV infection is defined based on a positive rapid test (≥12 months of age), or positive HIV DNA PCR (<12 months). HIV exposure is defined as a positive maternal HIV rapid test. Nutritional status is assessed using WHO standards, where severe acute malnutrition (SAM) is defined as anyone of weight-for-height Z-score (WHZ)<-3 SD from WHO standard, mid-upper-arm circumference (MUAC)<115 mm, or nutritional oedema [22]. Within the surveillance platform, a nested case-control study identified children with AGE who were age-eligible for vaccination and produced a stool sample that tested positive for rotavirus on Enzyme Immune Assay (EIA) as cases, and rotavirus test-negative controls.

### 2.1 Laboratory methods

Stool samples were batch-tested weekly for rotavirus antigen using enzyme immunoassay (EIA, Rotaclone, Meridian Bioscience, Cincinnati, Ohio). HIV testing of mothers and children was conducted using two sequential EIA rapid tests (Determine HIV-1/2 [Abbott Laboratories, USA] and Uni-Gold HIV [Trinity Biotech PLC, Ireland]), or HIV DNA PCR for infants under one year of age [20].

### 2.2 Statistical analysis

#### 2.2.1 Descriptive analysis

Continuous normally distributed data were described using mean and standard deviation (SD), or median and interquartile range otherwise. Differences in independent categorical covariates were assessed using χ^2^ tests. Student’s t or rank sum tests were used to compare independent means or medians, respectively.

#### 2.2.2 Prevalence changes over time of EIA-positive rotavirus

To evaluate yearly differences in rotavirus prevalence since vaccine introduction, we analysed the surveillance platform as a cohort and used Poisson regression models with robust standard errors [23] to estimate the relative risk of rotavirus in hospitalised AGE cases compared to the year preceding introduction. Models were adjusted for age and month of presentation, and analysis restricted to the first 6 months (January to June) of each year for consistency.

#### 2.2.3 Time series analysis

We used time series analysis to describe trend and seasonality in the prevalence of rotavirus in diarrhoeal stools over time. We applied a locally weighted smoother (defined as (1/8) ∗ [1 ∗ x(t − 2) + 2 ∗   x(t − 1) + 2 ∗ x(t) + 2 ∗ x(t + 1) + 1 ∗ x(t + 2)]) to mean monthly proportion of rotavirus-positive stools to define seasonality and a second locally weighted smoother (defined as (1/24) ∗ [1 ∗ x(t − 6) + 2 ∗ x(t-5) + 2 ∗ x(t − 4) + 2 ∗ x(t − 3) + 2 ∗ x(t − 2) + 2 ∗ x(t − 1) + 2 ∗ x(t) + 2 ∗ x(t + 1) + 2 ∗ x(t + 2) + 2 ∗ x(t + 3) + 2 ∗ x(t + 4) + 2 ∗ x(t + 5) + 1 ∗ x(t + 6)], where x(t) = percentage rotavirus-positive stools per month) to the same to define secular trend. Trend in rotavirus prevalence over time was then assessed in a linear model.

#### 2.2.4 Case-control analysis for vaccine effectiveness

Vaccine effectiveness (VE) was estimated from the nested case-control study as (1-Odds Ratio for 2 vs 0-dose vaccine receipt) among rotavirus EIA-positive rotavirus cases against EIA test-negative gastroenteritis controls, defined as described above, using unconditional logistic regression, and reported as a percentage. Estimates were adjusted for age and for secular and seasonal fluctuations using indicator variables for year and month of admission.

#### 2.2.5 Estimating indirect vaccine effects

Poisson regression models with robust standard errors were used to identify any change in the prevalence of rotavirus-positive gastroenteritis among unvaccinated admitted infants (<12 months of age) and children (12–59 months of age) following introduction of rotavirus vaccine using all available data. We then estimated the expected overall reduction in incidence of rotavirus gastroenteritis among admitted infants based on the direct effect of vaccination alone and compared this to the observed overall reduction. We made the assumption that any additional observed reduction in incidence may be attributed to the indirect effects of the vaccine [24]. Because we did not have data for a full calendar year prior to rotavirus vaccine introduction, incidence estimates were calculated for the first 6 months of each year. This was done using the following method [24]:

i. Incidence (case numbers per 100,000 age-specific population per 6 months) was estimated using projected population estimates for Blantyre city, based on the proportion of infants at the national level.
ii. The expected population direct effect (assuming any reduction in the overall incidence was due only to the direct effect of vaccination) was calculated as vaccine coverage × vaccine efficacy,
where both vaccine coverage and efficacy were expressed as proportions and using a value of vaccine efficacy of 49.4% for severe disease in infants, 34.5% for all rotavirus gastroenteritis in infants and 17.6% for children with severe disease [6], [7]. No efficacy estimates were available for disease of all severity in children, so expected effects were not calculated for this group.
iii. The observed overall effect was calculated by comparing the semi-annual post-vaccination incidence to incidence for the half-year prior to vaccine introduction (January to June 2012) using:
(pre-vaccine incidence – post-vaccine incidence)/pre-vaccine incidence.
iv. The estimated indirect effect was calculated as *observed effect – expected effect* [24].

For comparison purposes and to look for evidence of secular trends in diarrhoeal admissions to QECH, the incidence rate of hospitalised rotavirus-negative gastroenteritis was estimated using the denominators described above to estimate child-years at risk, and change over time was evaluated using incidence rate ratios.

Analyses were restricted to those children with stool samples collected. All analyses except VE estimates were restricted to admitted children. For analysis of trend in rotavirus prevalence and VE estimates, children were categorised into <12 month or 12–23 month age groups. For assessment of vaccine indirect effects, children up to 59 months of age were included. Analyses were conducted using Stata 12.1 (™Statacorp, College Station, Texas USA).

### 2.3 Ethics

Ethical approval was obtained from the National Health Sciences Research Committee, Lilongwe, Malawi (867), and by the University of Liverpool Research Ethics Committee (000490).

## 3 Results

Stool samples were collected from a total of 2320 children (median age 10.68 months, interquartile range [IQR] 7.72, 15.29) between 1st January 2012 and 30 June 2016. Of these, 1318 (median age 10.04 months, IQR 7.59–13.63) were eligible for both doses of rotavirus vaccine, and 1130 (median age 9.74 months, IQR 7.43–12.75) had documented evidence of vaccine status. Characteristics of the population are shown in Table 1.

**Table 1:** Demographic data.

Characteristic		Denominator
Male (n, %)	1339 (57.77)	2318
Age in months (median, IQR)	10.68 (7.72, 15.29)	2320
Weight for height Z score (WHZ)* (mean, SD)	−0.85 (1.89)	2292
Severe acute malnutrition (n, %)*	396 (17.31)	2288

RV coverage** (n, %)		
0 doses	43 (3.81)	1130
1 dose	60 (5.31)	1130
2 doses	1027 (90.88)	1130

HIVϮ		
Infected (n, %)	71 (4.03)	1761
Exposed (n, %)	426 (18.80)	2266

* Weight corrected by adding 10% to weights for those with severe disease to account for dehydration.

** In those vaccine age-eligible with health record confirmation.

Ϯ HIV infected is defined as a positive HIV rapid test over 12 months of age, or a positive HIV DNA PCR result. HIV exposed is defined as a positive maternal HIV rapid test.

### 3.1 Overall decline in rotavirus prevalence

**Fig. 1:**
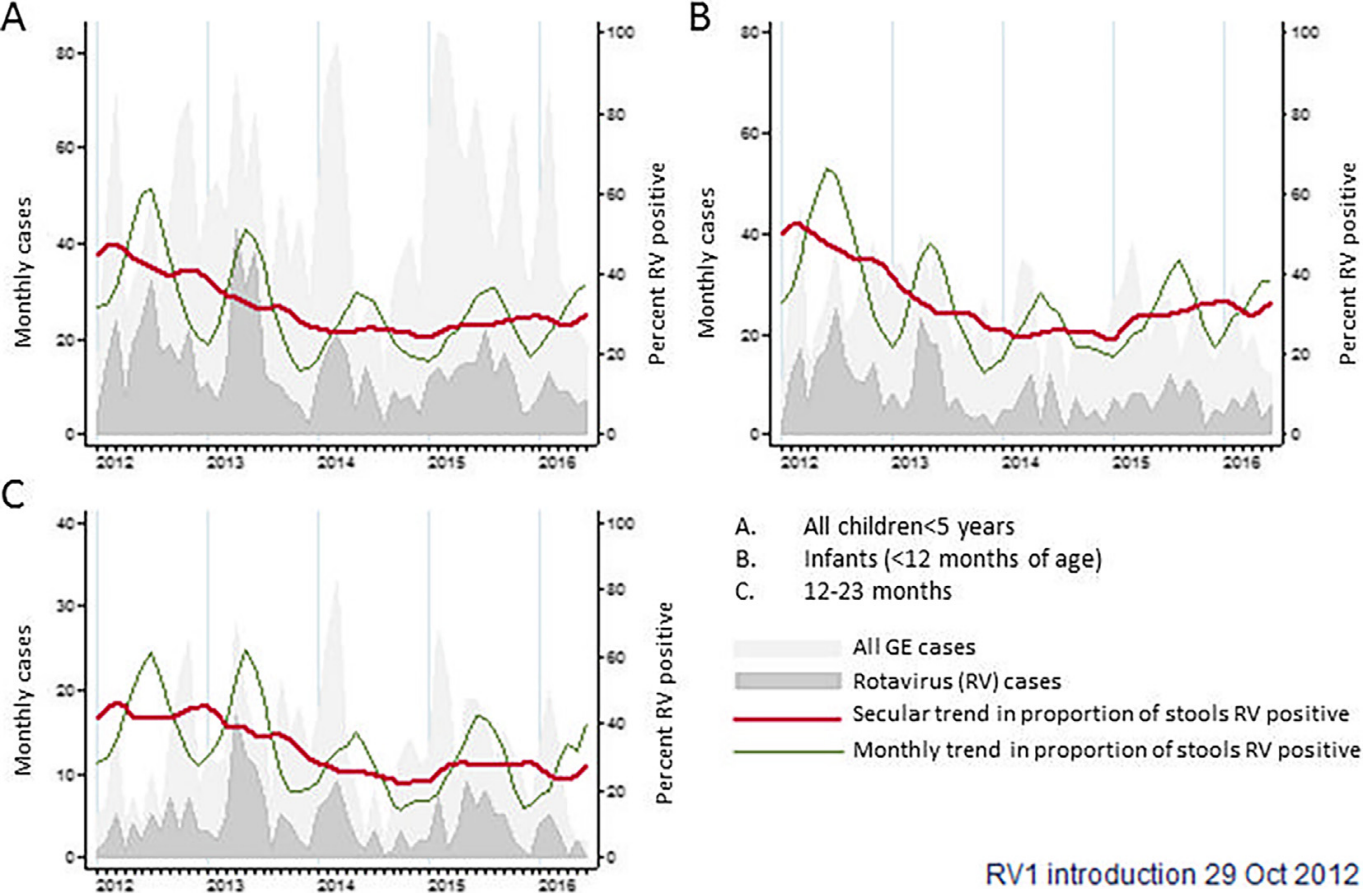
Monthly diarrhoeal admissions to QECH.

In the four years since vaccine introduction, the relative risk of rotavirus among children admitted with diarrhoeal disease has consistently declined (Table 2 and Fig. 1); however, over 25% of all gastroenteritis admissions remain rotavirus positive (Table 2). The median age of cases has increased from 9.48 (IQR 7.00, 13.54) months prior to vaccine introduction to 10.86 (IQR 7.95, 15.41) months following vaccine introduction (rank sum test p < 0.001). The adjusted relative risk of rotavirus positivity among infants hospitalised with gastroenteritis in the first 6 months of the year has decreased from 69/139 [49.64%] to 197/607 [32.45%] since vaccine introduction (adjusted RR 0.67 [95% CI 0.55, 0.82] p < 0.001). This effect is smaller in children aged 12–23 months, where the relative risk pre- and post-introduction respectively was 15/37 (40.54%) and 122/352 (34.66%) (adjusted RR 0.85 [95% CI 0.57, 1.28] p = 0.440) (Fig. 1). The proportion of admitted rotavirus-positive cases aged 12–23 months increased from 15/84 (17.86%) in the January to June prior to vaccine introduction to 122/319 (38.24%) subsequent to vaccine introduction (χ^2^p<0.001). Linear regression showed a significant negative trend in prevalence of rotavirus over time in infants (regression coefficient −0.35 [95% CI −0.46, −0.25] P < 0.001) and in children aged 12–23 months (regression coefficient −0.43 [95% CI −0.51, −0.36] p < 0.001), where the regression coefficient represents the percentage change in rotavirus positivity per month.

**Table 2:** Relative risk of rotavirus detection in children admitted to QECH with gastroenteritis.

	RV** negative	RV positive	Total	RR (95% CIϮ)*
*Time period*				
Pre-vaccine (Jan’12-Jun’12)	110 (56.70)	84 (43.30)	194	1 (REF)
Jan’13-Jun’13	185 (58.18)	133 (41.82)	318	0.95 (0.78–1.16)
Jan’14-Jun’14	177 (69.96)	76 (30.04)	253	0.77 (0.61–0.98)
Jan’15-Jun’15	219 (75.26)	72 (24.74)	291	0.60 (0.46–0.77)
Jan’16-Jun’16	132 (72.13)	51 (27.87)	183	0.74 (0.57–0.98)

Total	823 (66.42)	416 (33.58)	1239	

* Adjusted for age in months and month at admission. Relative risk for rotavirus gastroenteritis vs test-negative gastroenteritis.

Ϯ 95% confidence interval.

** Rotavirus (RV).

### 3.2 Vaccine effectiveness estimates

The adjusted VE for two doses of vaccine, across all ages and disease severity was 61.89% (95% CI 28.04%, 79.82%) (Table 3), whilst VE against all disease in the 12–23 month age group was 31.69% (95% CI −139.03%, 80.48%). VE against severe disease among children <12 months of age was 83.24% (95% CI 53.81%, 93.92%).

**Table 3:** Vaccine effectiveness estimates.

	N (Rotavirus positive, vaccinated [%])**	N (Rotavirus positive unvaccinated [%])**	Vaccine effectiveness (95% CI)	P value
Adjusted*				
2 doses	275/1019 (26.99)	20/43 (46.51)	61.89 (28.04–79.82)	0.003

Disease severity* (all ages)				
Severe	243/788 (30.84)	15/25 (60.00)	74.75 (41.49–89.10)	0.001
Mild/mod	30/214 (14.02)	4/17 (23.53)	25.81 (−165.58–79.27)	0.646

By age* (all severity)				
<12 m	190/696 (27.30)	16/29 (55.17)	74.88 (44.59–88.61)	0.001
12–23 m	78/285 (27.37)	4/13 (30.77)	31.69 (−139.03–80.48)	0.551

By age* (severe disease)				
<12 m	166/547 (30.35)	13/19 (68.42)	83.24 (53.81–93.92)	0.001
12–23	71/215 (33.02)	2/6 (33.33)	7.58 (−444.91–84.33)	0.931

* Adjusted for age, and year and month of presentation. All are two-dose estimates.

** Where N refers to the number of rotavirus positive cases; denominator is all gastroenteritis cases with stool sample collected, vaccinated indicates 2 doses of monovalent rotavirus vaccine received and unvaccinated indicates 0 doses.

### 3.3 Indirect vaccine effects

**Fig. 2:**
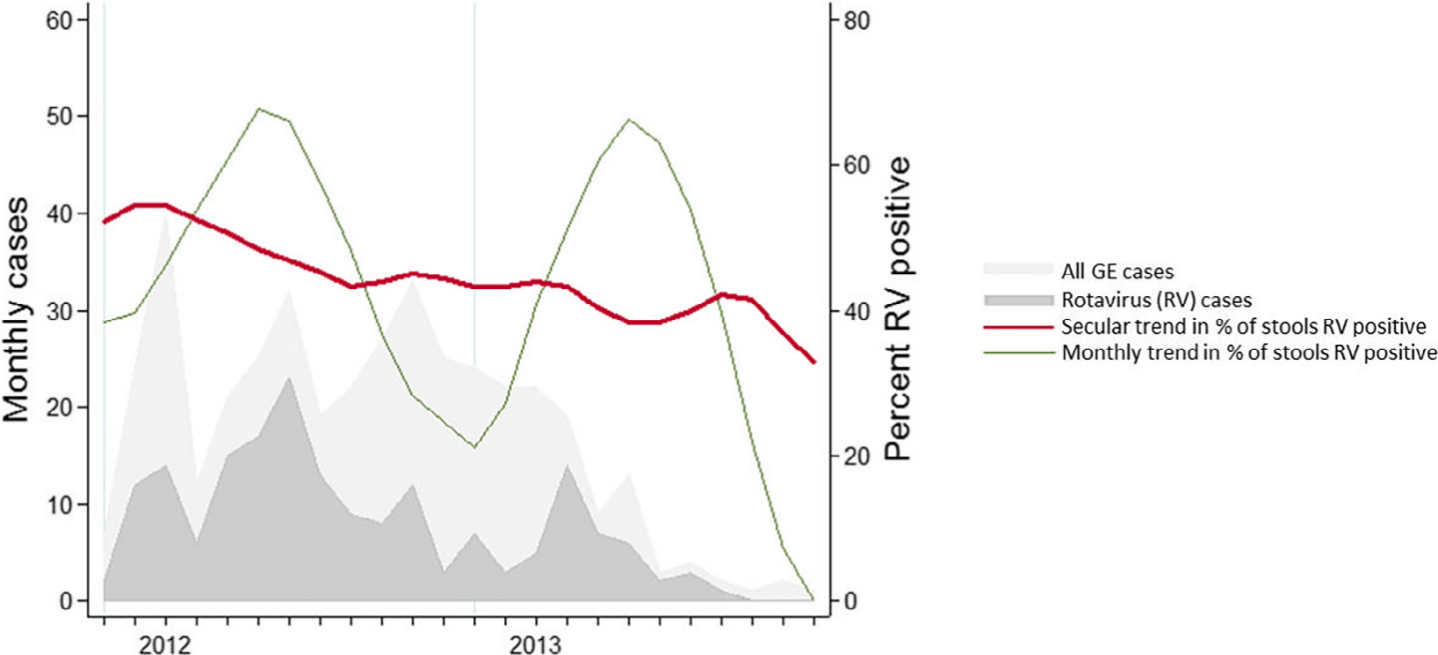
Monthly diarrhoeal admissions to QECH in unvaccinated infants – note data truncated at 24 months from start of surveillance (14 months from vaccine introduction) due high vaccine coverage.

Among unvaccinated infants with gastroenteritis, rotavirus prevalence declined from 117/221 (52.94%) in the 10 months prior to vaccine introduction to 65/184 (35.33%) in the 14 months following vaccine introduction (adjusted RR 0.70 [95% CI 0.55, 0.88] p = 0.003) (Fig. 2). This analysis was truncated at 24 months from the start of surveillance because the vast majority of infants after this time-point were vaccinated. Comparing the observed against expected reduction in incidence showed a difference of between 9 and 24% in admitted infants with rotavirus gastroenteritis of any severity (Table 4). Restricting to infants with severe rotavirus gastroenteritis, no difference was seen (Table 4). In unvaccinated children 12–59 months of age there was no evidence of a decline in the prevalence of rotavirus following vaccine introduction, with 26/84 (30.95%) rotavirus positive pre-vaccine introduction and 70/193 (36.27%) rotavirus positive after introduction, RR 1.14 (95% CI 0.79, 1.63). This analysis was restricted to 36 months after the start of surveillance. There was also no evidence of an indirect effect demonstrated on comparison of observed vs expected reductions in incidence (Table 4).

**Table 4:** Comparison of expected and observed vaccine effects by year since vaccine introduction.

	Incidence*	RV coverage** (%)	Expected effect (%)	Observed effect (%)	Difference in observed effect (%)
*<12 m severe RV GE*					
Jan’12-Jun’12Ϯ	183	–	–	–	–
Jan’13-Jun’13	210	29.60	14.62	−14.83	−29.45
Jan’14-Jun’14	110	92.93	45.91	39.75	−06.15
Jan’15-Jun’15	116	94.16	46.52	36.22	−0.10
Jan’16-Jun’16	99	94.29	46.58	45.92	−0.66
All post vaccine	134	76.61	37.85	26.76	−11.08

*<12 m all RV GE*					
Jan’12-Jun’12 Ϯ	234	–	–	–	–
Jan’13-Jun’13	256	29.41	10.21	−9.22	−19.43
Jan’14-Jun’14	139	90.08	31.26	40.73	9.47
Jan’15-Jun’15	123	94.32	32.73	47.45	14.73
Jan’16-Jun’16	102	92.31	32.03	56.39	24.13
All post vaccine	155	73.16	25.39	33.84	8.45

*12–60 m severe RV GE*					
Jan’12-Jun’12 Ϯ	14	–	–	–	–
Jan’13-Jun’13 Ϯ Ϯ	58	–	–	–	–
Jan’14-Jun’14	26	64.00	11.26	−79.71	−90.98
Jan’15-Jun’15	26	90.77	15.98	−81.28	−97.26
Jan’16-Jun’16	14	95.12	16.74	−0.30	−17.04
All post vaccine	22	85.17	14.96	−53.76	−68.73

* 6 month Jan to June, per 100,000 children.

** % coverage for 2nd dose of rotavirus vaccine.

Ϯ Prior to vaccine introduction.

Ϯ Ϯ Period from Jan’13 to Jun’13 excluded for children over 12 months as these children were not vaccine age eligible.

In contrast to the incidence rate of rotavirus-positive hospitalisations, the incidence of hospitalisation for rotavirus-negative gastroenteritis increased over time. The incidence rate of hospitalisation with rotavirus-negative gastroenteritis among infants was 479 per 100,000 child-years at risk prior to rotavirus vaccine introduction and 655 per 100,000 child-years at risk subsequently (incidence rate ratio 1.37 [95% CI 1.06, 1.79]). For children, the incidence rate for hospitalisation with rotavirus-negative gastroenteritis was 77 per 100,000 child-years pre- and 136 per 100,000 child-years post-rotavirus-vaccine introduction (incidence rate ratio 1.77 [95% CI 1.27, 2.52]).

## 4 Discussion

Four years after programmatic rotavirus vaccine implementation in Malawi, we continue to demonstrate VE against rotavirus disease of all severity (VE 61.89%, 95% CI 28.04–79.82%), and a consistent decline in the prevalence of rotavirus in children presenting to hospital with gastroenteritis. There is some evidence of a reduction in disease among unvaccinated infants, possibly attributable to indirect vaccine effects. However, children aged 12–23 months demonstrate a less pronounced reduction in disease and a lower point estimate for VE compared to those under 12 months of age, and overall there remains a significant residual burden of disease with rotavirus associated with just over 1 in 4 gastroenteritis admissions.

Gavi support has allowed LMICs such as Malawi to successfully introduce rotavirus vaccination. However, in the long-term immunisation programmes must be locally funded. Robust vaccine effectiveness estimates, demonstration of sustained impact, and evaluation of indirect effects are essential to allow national governments to evaluate the cost-effectiveness of vaccines. A major strength of our study is its basis in a long-standing surveillance system, within which a case-control study to evaluate vaccine effectiveness has been nested. As vaccine coverage increases, and as it becomes increasingly difficult to identify unvaccinated children for the case-control study, on-going surveillance will allow evaluation of incidence over time. The high vaccine effectiveness seen in infants with severe disease (83.24% [95% CI 53.81%, 93.92%] is striking. Although this result should be interpreted within the context of moderately wide confidence limits, it is consistent with our previously reported estimates for Malawi and with recent estimates for RV5 from Rwanda [25], and together with the demonstration of robust vaccine effectiveness against rotavirus disease of any severity is encouraging evidence of real-world value of rotavirus vaccines in preventing disease in low-income countries.

Effectiveness point estimates in children aged 12–23 months appear lower than those in infants. The confidence bounds around these estimates are wide, and it is possible that this simply reflects uncertainty in point estimates as a result of fewer AGE episodes in the older age group, but the finding is corroborated by the relative risk of rotavirus gastroenteritis compared to test-negative controls, which has not decreased in this age group, and the proportion of rotavirus cases occurring in children 12–23 months of age, which has increased from 18% to 38% following vaccine introduction. This lower VE in the second year of life is consistent with observations in Colombia and Brazil [26], [27], but inconsistent with South Africa and Botswana [15], [17]. While it is possible that the lower VE is due to immunological waning (indeed a randomised controlled trial (RCT) in Malawi showed a non-significantly higher vaccine efficacy in the second year of life among infants given a three-dose RV1 schedule [7], [28]), our findings could also be explained by an epidemiological phenomenon. A high force of rotavirus infection in countries like Malawi results in unvaccinated children experiencing frequent wild-type rotavirus exposure over time, providing natural immunity even among the unvaccinated. This effect could then lead to a reduction in observed VE in the second year of life as time for wild-type exposure to occur has accumulated.

Indirect effects of rotavirus vaccine have been observed in high-income countries [18], but there is currently little evidence from LMICs [13]. We observed reductions in the relative risk of rotavirus-positive gastroenteritis in unvaccinated infants in the year following vaccine introduction, though the reliability of this estimate is limited by the short duration of observation. Available data across all months were included for this analysis to maximise data availability, acknowledging the possibility that this could introduce some seasonal bias. Sensitivity analysis restricting to the first 6 months of the year resulted in similar observations to the full period analysis, but the results were no longer significant. Comparison of observed with expected overall reductions in rotavirus incidence showed greater reductions than would be explained by direct effects alone in infants with disease of any severity. However, this effect was not observed among infants with severe disease or among children 12–59 months of age.

The absence of observed indirect effects in these groups is contrary to findings from higher income settings and from Rwanda [13], [18], but supported by studies from Zambia and South Africa which found no evidence of a rotavirus vaccine indirect effect in children too old to receive vaccine [29], [30]. The absence of identifiable indirect effects in infants with severe disease could potentially be explained by the larger vaccine efficacy estimate in this group. One explanation for the absence of identifiable indirect effect in children over 12 months of age could be that prior to vaccine introduction, older children in Malawi experienced a low frequency of clinical disease because of frequent natural exposure to rotavirus and consequent natural immunity. A reduction in transmission in the time-period immediately following vaccine introduction may therefore confer no additional protection.

These findings could however also represent under-ascertainment by this study. In particular, our measurement of indirect effects only captures the effect on hospitalised cases which are a small proportion of the community burden of rotavirus disease, and the observed increase in incidence of hospitalisation with rotavirus-negative diarrhoeal disease following vaccine introduction could bias estimation of vaccine impact based on hospitalised cases and estimation of indirect effects derived from this towards the null. Increasingly high vaccine coverage limits further analysis of indirect effects, and mathematical modelling may be required to investigate the presence and extent of such effects in greater depth.

It is not clear why we observed an increase in incidence of test-negative diarrhoeal cases following vaccine introduction. This could represent secular changes in health-care seeking behaviour or admission patterns with time, but also may reflect the short period of enhanced surveillance before this point, which limits assessment of trends in rotavirus prevalence prior to vaccine introduction. Prevalence estimates can be affected by secular changes in denominators, and ongoing surveillance is required to evaluate the long term impact of vaccine in our setting.

## 5 Conclusions

Our data demonstrate substantial and consistent reductions in rotavirus prevalence in hospitalised children four years since vaccine introduction and robust vaccine effectiveness against all-severity rotavirus disease in infants reinforcing the importance of rotavirus vaccine in reducing the burden of disease in low-income countries. Additionally our data suggest the presence of population indirect effect in infants, although not in other age groups. Although factors other than vaccine introduction may also contribute to overall disease reduction, our analysis was not designed to formally account for these. We are however, unaware of major health system or socioeconomic or nutritional changes in Malawi over the period of our observation that might have also contributed to reductions in disease incidence. Importantly, our data also show the persistent burden of rotavirus, particularly in children in the second year of life. Given the ongoing prevalence of rotavirus disease in this population, strategies to further improve the effectiveness of current vaccines maybe required to maximise protection for all children against rotavirus disease.
